# Supplementary material for: Heavy Metals Enrichment Associated with Water-Level Fluctuations in the Riparian Soils of the Xiaowan Reservoir, Lancang River
Source: Int J Environ Res Public Health. 2022 Oct 8;19(19):12902. doi: 10.3390/ijerph191912902 (PMC9566251; doi:10.3390/ijerph191912902)
Supplement: Supplementary file 1 [file ijerph-19-12902-s001.zip › ijerph-1921015-supplementary.pdf]

Supplementary Materials

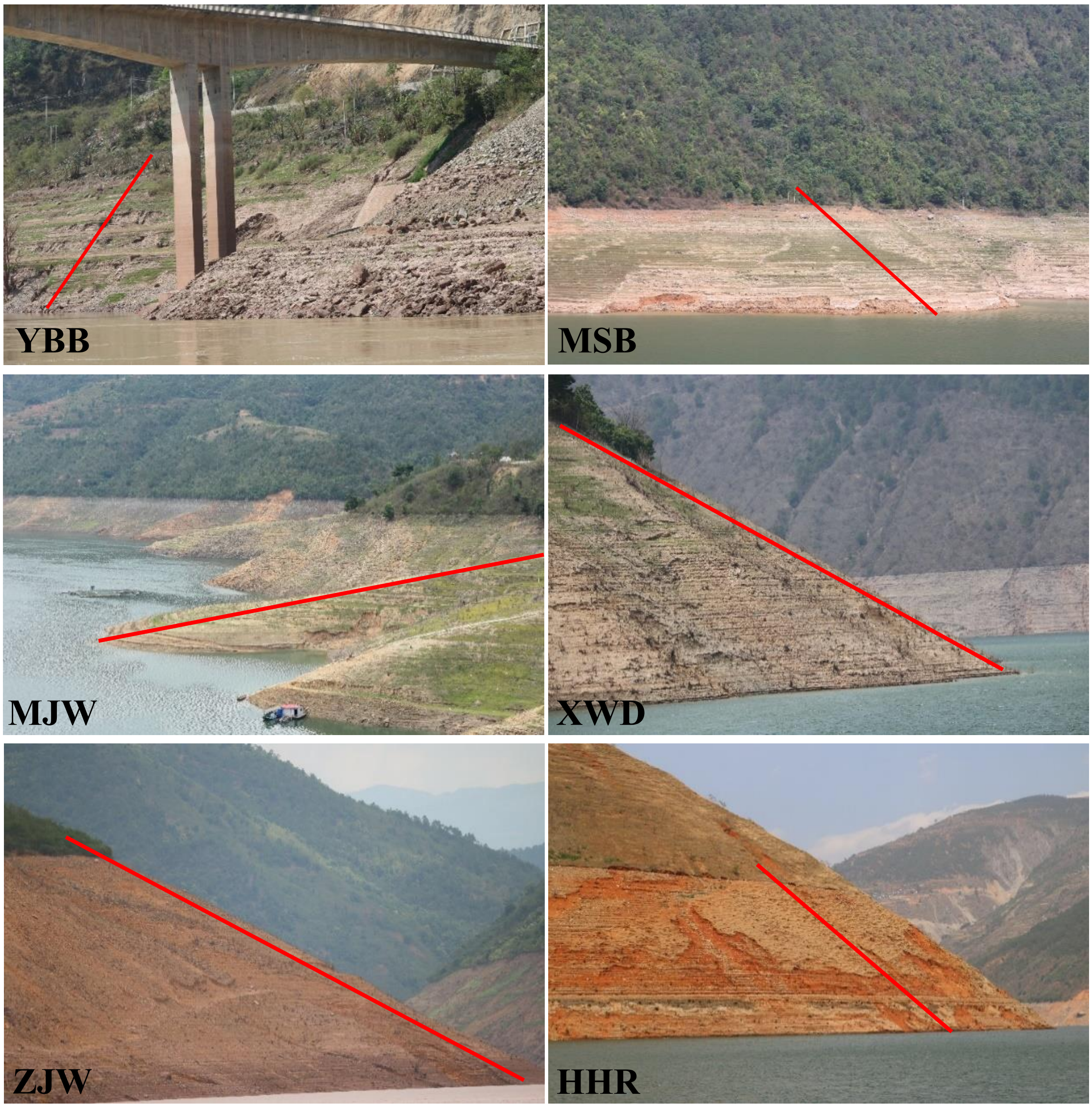

Figure S1. The riparian soils sampling locations of Xiaowan Reservoir

Table S1. The statistics of the heavy metal concentrations in the riparian soils of XWR.

**Element (mg/kg)**	**As**	**Cd**	**Cr**	**Cu**	**Hg**	**Ni**	**Pb**	**Zn**
**(a) YBB (*n* = 6)**								
Maximum	8.30	0.09	71.00	49.40	0.03	45.90	14.70	62.00
Minimum	2.00	0.05	57.00	39.20	0.01	37.80	8.90	36.00
Mean*	5.08 ± 0.81^{c}	0.07 ± 0.00^{a}	**67.00 ± 1.89**^{ac}	**43.72 ± 1.39^{a}**	0.02 ± 0.00^{b}	**42.08 ± 1.10^{ab}**	11.57 ± 0.85^{f}	51.00 ± 3.38^{e}
Std. D	1.98	0.01	4.62	3.42	0.01	2.68	2.07	8.29
**IRZ**	6.6	0.054	57	39.2	0.03	44.5	12.7	49
**(b) MSB (*n* = 10)**								
Maximum	3.20	0.14	41.00	16.00	0.03	13.70	23.80	98.00
Minimum	1.70	0.07	29.00	9.50	0.01	7.90	20.90	67.00
Mean*	2.53 ± 0.15^{cf}	0.10 ± 0.00^{f}	33.50 ± 1.13^{e}	12.71 ± 0.66^{be}	0.02 ± 0.00^{b}	10.70 ± 0.49^{f}	22.09 ± 0.27^{bc}	**81.60 ± 3.24^{ab}**
Std. D	0.48	0.02	3.58	2.10	0.01	1.55	0.86	10.24
**IRZ**	2.8	0.07	32	10.3	0.02	11.5	21.8	69
**(c) MJW (*n* = 14)**								
Maximum	29.40	0.07	91.50	28.70	0.05	40.10	56.70	116.50
Minimum	6.10	0.02	37.00	8.10	0.01	17.60	32.10	48.00
Mean*	**12.69 ± 1.75^{b}**	0.03 ± 0.00^{e}	**69.79 ± 4.85^{ab}**	15.44 ± 1.48^{bd}	0.02 ± 0.00^{b}	30.66 ± 1.97^{cd}	**39.43 ± 1.49^{a}**	72.14 ± 4.82^{ad}
Std. D	6.55	0.01	18.17	5.53	0.01	7.36	5.59	18.04
**IRZ**	17.7	0.07	82	22.4	0.02	38	39.1	97
**(d) XWD (*n* = 14)**								
Maximum	3.80	0.08	66.00	9.60	0.03	31.50	9.60	40.00
Minimum	1.70	0.02	48.00	2.40	0.01	17.30	7.00	19.00
Mean^{a}	2.46 ± 0.15^{ce}	0.04 ± 0.00^{a}	55.79 ± 1.12^{cd}	4.83 ± 0.51^{f}	0.02 ± 0.00^{b}	23.61 ± 0.85^{e}	8.06 ± 0.19	25.07 ± 1.65^{f}
Std. D	0.55	0.02	4.19	1.90	0.01	3.19	0.72	6.16
**IRZ**	2.7	0.03	48	9.6	0.01	17.3	7.3	27
**(e) ZJW (*n* = 14)**								
Maximum	58.50	0.25	73.00	22.20	0.30	37.40	34.40	134.00
Minimum	37.10	0.02	48.00	15.40	0.05	24.80	17.10	50.00
Mean*	**49.21 ± 1.50^{a}**	0.06 ± 0.01^{a}	56.07 ± 1.75^{c}	18.66 ± 0.56^{bc}	**0.13 ± 0.02^{a}**	30.81± 0.95^{c}	23.01 ± 1.11^{b}	74.36 ±5.85^{ac}
Std. D	5.61	0.06	6.54	2.10	0.09	3.54	4.17	21.89
**IRZ**	37.1	0.02	56	18.6	0.06	25.8	17.6	52
**(f) HHR (*n* = 14)**								
Maximum	4.80	0.09	96.00	30.30	0.03	55.40	27.40	100.00
Minimum	2.90	0.02	74.00	16.20	0.02	33.60	17.60	74.00
Mean*	3.69 ± 0.17^{cd}	0.05 ± 0.01^{a}	**89.57 ± 1.34^{a}**	23.57 ± 1.21^{b}	0.02 ± 0.00^{b}	**44.84 ± 1.41^{a}**	20.81 ± 0.65^{bd}	**86.79 ± 2.44^{a}**
Std. D	0.63	0.02	5.02	4.52	0.00	5.27	2.44	9.13
**IRZ**	3.516.96	0.0541.92	945.61	30.319.16	0.0216.33	47.811.74	27.411.72	10010.52

* Represented as a mean ± standard error. Data in the bold text indicates the exceeded the background value of Yunnan Provence. Small letters in the upper right of "Mean*" value represent the significant difference of trace metals concertation along different sampling sections
